# Supplementary material for: Regression-Based Normative Data for Independent and Cognitively Active Spanish Older Adults: Free and Cued Selective Reminding Test, Rey–Osterrieth Complex Figure Test and Judgement of Line Orientation
Source: Int J Environ Res Public Health. 2021 Dec 9;18(24):12977. doi: 10.3390/ijerph182412977 (PMC8701853; doi:10.3390/ijerph182412977)
Supplement: Supplementary file 1 [file ijerph-18-12977-s001.zip › ijerph-1440901-supplementary.pdf]

International Journal of
Environmental Research and Public Health

MDPI

Supplementary Material

Regression-Based Normative Data for Independent and Cognitively Active Spanish Older Adults: Free and Cued Selective Reminding Test, Rey-Osterrieth Complex Figure Test and Judgement of Line Orientation

Eva Calderón-Rubio [1], Javier Oltra-Cucarella [1,2,*], Beatriz Bonete-López [1,2], Clara Iñesta [1] and Esther Sitges-Maciá[1,2]

Table S1. Comparing number of low scores on FCSRT-Imm between normative data sets (NEURONORMA and SABIEX).

		SABIEX		
		0	1+	Total
NEURONORMA	0	94	7	101
	1+	0	2	2
Total		94	9	103

FCSRT-Imm: Free and Cued Selective Reminding Test, immediate recall.

Table S2. Comparing number of low scores on FCSRT-Del between normative data sets (NEURONORMA and $SABIEX_{INDEP}$).

		SABIEX		
		0	1+	Total
NEURONORMA	0	91	11	102
	1+	0	1	1
Total		91	12	103

FCSRT-Del: Free and Cued Selective Reminding Test, delayed recall.

Table S3. Comparing number of low scores on FCSRT-Del independent (NEURONORMA) with FCSRT-Del conditional on FCSRT-Imm ($FCSRT\text{-}Del_{SABIEX}$).

		SABIEX		
		0	1+	Total
NEURONORMA	0	91	11	102
	1+	0	1	1
Total		91	12	103

FCSRT-Del: Free and Cued Selective Reminding Test, delayed recall.

Table S4. Comparing number of low scores on ROCF-Imm between normative data sets (NEURONORMA and SABIEX$_{INDEP}$).

		SABIEX		
		**0**	**1+**	**Total**
**NEURONORMA**	**0**	91	5	96
	**1+**	1	6	7
**Total**		92	11	103

ROCF-Imm: Rey-Osterrieth Complex Figure, immediate recall.

Table S5. Comparing number of low scores on ROCF-Imm independent (NEURONORMA) with ROCF-Imm conditional on ROCF-C (ROCF-Imm$_{SABIEX}$).

		SABIEX		
		**0**	**1+**	**Total**
**NEURONORMA**	**0**	91	5	96
	**1+**	3	4	7
**Total**		94	9	103

ROCF-Imm: Rey-Osterrieth Complex Figure, immediate recall; ROCF-C: Rey-Osterrieth Complex Figure, copy trial.

Table S6. Comparing number of low scores on ROCF-Del between normative data sets (NEURONORMA and SABIEX$_{INDEP}$).

		SABIEX		
		**0**	**1+**	**Total**
**NEURONORMA**	**0**	89	4	93
	**1+**	1	9	10
**Total**		90	13	103

ROCF-Del: Rey-Osterrieth Complex Figure, delayed recall.

Table S7. Comparing number of low scores on ROCF-Del independent (NEURONORMA) with ROCF-Del conditional on ROCF-Imm (ROCF-Del$_{SABIEX}$).

		SABIEX		
		**0**	**1+**	**Total**
**NEURONORMA**	**0**	82	11	93
	**1+**	10	0	10
**Total**		92	11	103

ROCF-Del: Rey-Osterrieth Complex Figure, delayed recall; ROCF-Imm: Rey-Osterrieth Complex Figure, immediate recall.
